# Supplementary material for: A new juvenile Yamaceratops (Dinosauria, Ceratopsia) from the Javkhlant Formation (Upper Cretaceous) of Mongolia
Source: PeerJ. 2022 Apr 5;10:e13176. doi: 10.7717/peerj.13176 (PMC8992648; doi:10.7717/peerj.13176)
Supplement: Supplemental Information 1 [file peerj-10-13176-s001.docx]

Supplementary Information for

**A new juvenile *Yamaceratops* (Dinosauria, Ceratopsia) from the Javkhlant Formation (Upper Cretaceous) of Mongolia**

Minyoung Son^1,2^, Yuong-Nam Lee^1,*^, Badamkhatan Zorigt^2^, Yoshitsugu Kobayashi^3^, Jin-Young Park^1^, Sungjin Lee^1^, Su-Hwan Kim^1^, Kang-Young Lee^4^

^1^School of Earth and Environmental Sciences, Seoul National University, Seoul, South Korea

^2^Department of Earth and Environmental Sciences, University of Minnesota, Minneapolis, United States

^3^Institute of Paleontology, Mongolian Academy of Sciences, Ulaanbaatar, Mongolia

^4^Hokkaido University Museum, Hokkaido University, Sapporo, Japan

^5^Department of Physics Education, Gyeongsang National University, Jinju, South Korea

* Corresponding Author: Yuong-Nam Lee

1 Gwanak-ro, Gwanak-gu, Seoul National University, Seoul, 08826, South Korea

Email address: ynlee@snu.ac.kr

**This supplementary information file includes:**

**1. Supplementary images of MPC-D 100/553**

Figure S1. Ventral view of the snout of MPC-D 100/553.

Figure S2. Maxillary teeth of MPC-D 100/553.

Figure S3. Pedal ungual III of MPC-D 100/553

**2. Measurements of MPC-D 100/553**

Table S1. Measurements of the skull of *Yamaceratops dorngobiensis*, MPC-D 100/553

Table S2. Measurements of the axial elements of *Yamaceratops dorngobiensis*, MPC-D 100/553

Table S3. Measurements of the appendicular elements of *Yamaceratops dorngobiensis*, MPC-D 100/553

Table S4. Measurements of the left pes of *Yamaceratops dorngobiensis*, MPC-D 100/553

**3. Primary phylogenetic analysis**

Modified scoring of *Yamaceratops* for the Arbour and Evans (2019) matrix

Changes in scorings applied to the Arbour and Evans (2019) matrix

Ontogenetically variable characters from the Arbour and Evans (2019) matrix.

Time calibration from the Arbour and Evans (2019) matrix.

Figure S4. Phylogenetic relationships of *Yamaceratops dorngobiensis* among ceratopsians using the Arbour and Evans (2019) matrix with revised scorings, without pruning of taxa.

Figure S5. Phylogenetic relationships of *Yamaceratops dorngobiensis* among ceratopsians using the Arbour and Evans (2019) matrix with revised scorings, with *Helioceratops* removed.

**4. Supplementary phylogenetic analysis**

Modified scoring of *Yamaceratops* for the Yu et al. (2020) matrix

Changes in scoring for *Yamaceratops* applied to the Yu et al. (2020) matrix

Phylogenetic relationships of *Yamaceratops dorngobiensis* among ceratopsians using the Yu et al. (2020) matrix as modified from the Knapp et al. (2018) matrix

Figure S6. Phylogenetic relationships of *Yamaceratops dorngobiensis* among ceratopsians using the Yu et al. (2020) matrix

**5. Supplementary references**

**Other supplementary materials for this manuscript include three Supplementary files:**

Supplementary file 1. Character matrix by Arbour and Evans (2019) with *Yamaceratops* revised.

Supplementary file 2. Age data for time calibration of the Arbour and Evans (2019) matrix.

Supplementary file 3. Character matrix by Yu et al. (2020) with *Yamaceratops* revised.

**1. Supplementary images of MPC-D 100/553**


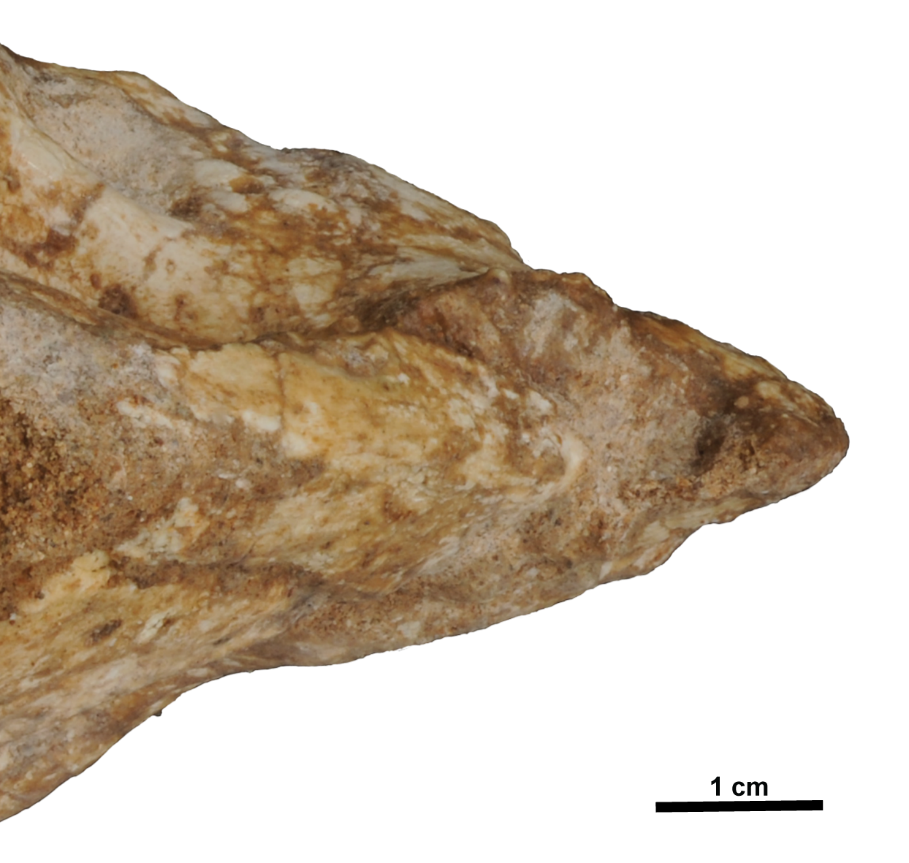


**Figure S1. Ventral view of the snout of MPC-D 100/553.** The bifurcate ventral process of the predentary is shown.

.


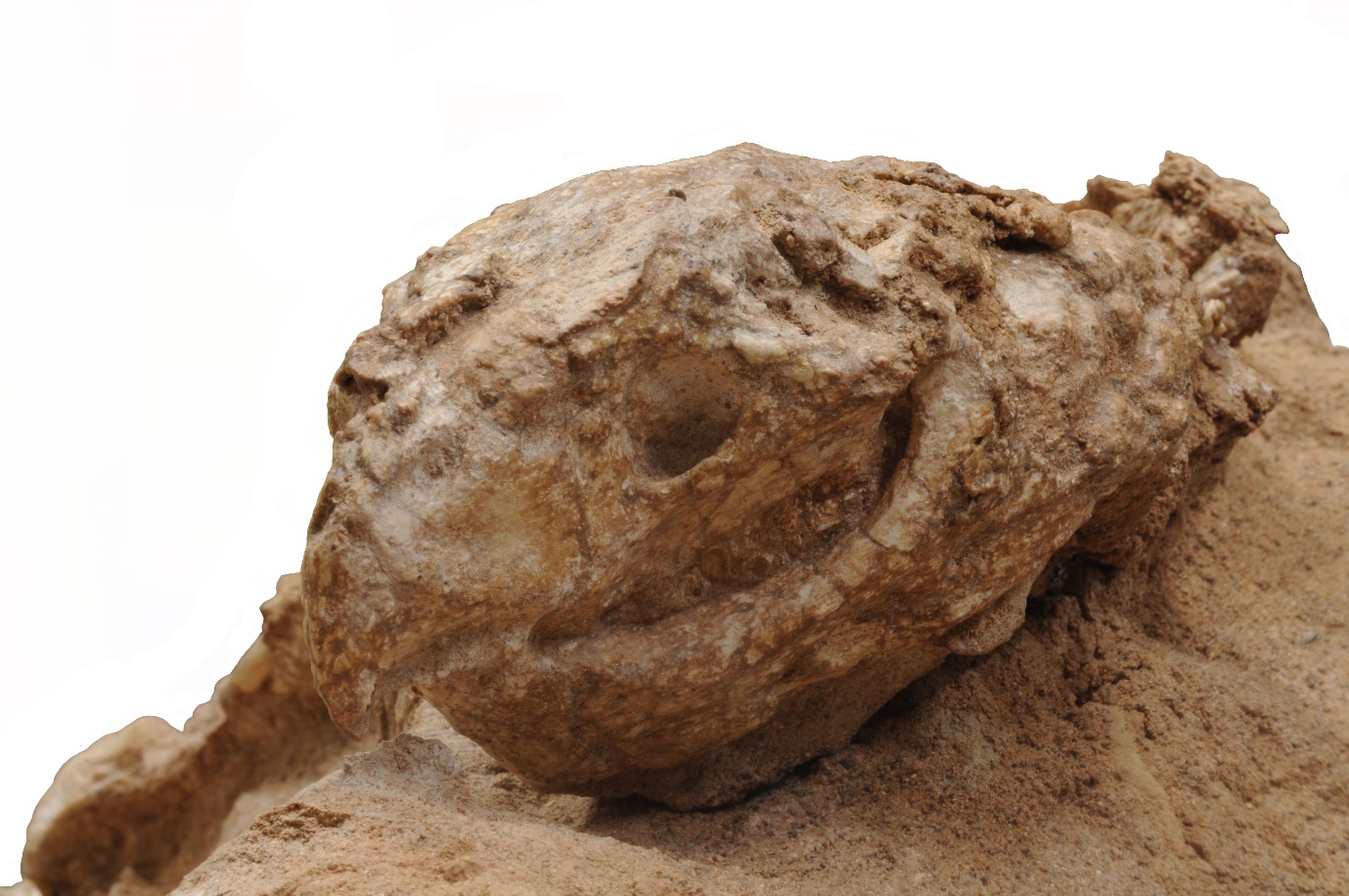


**Figure S2. Maxillary teeth of MPC-D 100/553.** The cingulum, along with primary and secondary ridges, is shown in left anterolateral view


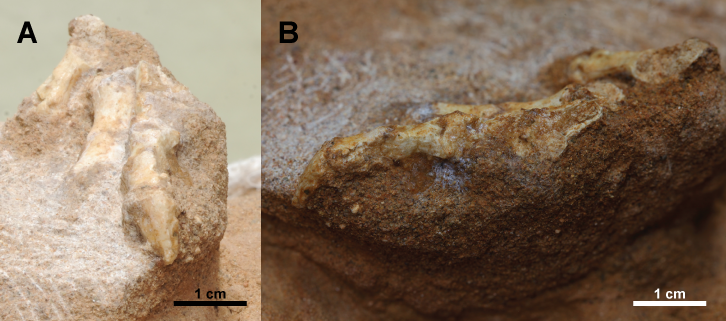


**Figure S3. Pedal ungual III of MPC-D 100/553.** (A) Pedal ungual III in dorsal profile view; (B) Pedal ungual III in left lateral view.

**2. Measurements of MPC-D 100/553**

**Table S1. Measurements of the skull of *Yamaceratops dorngobiensis*, MPC-D 100/553.** The standard measurement numbers follow Dodson (1976), except for 41, which followed Morschhauser et al. (2018a). An asterisk indicates measurement from a broken element as preserved.

| Element | Measurement number and description |  | Left | Right |
| --- | --- | --- | --- | --- |
| Skull | 1. Basal skull length (rostral to angular as preserved) | 110.65 | NA | 110.65 |
| Skull | 2. Total skull length (preserved) | >138.49 | NA | 138.49* |
| Skull | 3. Preorbital length | 46.715 | 47.09 | 46.34 |
| Skull | 4. Postorbital length | 47.44 | NA | 47.44 |
| Skull | 5. Frill length (preserved) | 45.66 | NA | 45.66 |
| Skull | 6. Skull width across maxillae | 27.11 | NA | NA |
| Skull | 7. Skull width across postorbitals | 40.72 | NA | 20.36 |
| Skull | 8. Skull width across jugals | 93.7 | NA | 46.85 |
| Skull | 10. Skull height nasal | 42.59 | 32.51* | 42.59 |
| Skull | 11. Skull height postorbital | 33.19 | NA | 33.19 |
| Orbit | 13. Orbit length | 33.69 | NA | 33.69 |
| Orbit | 14. Orbit height | 20.17 | NA | 20.17 |
| External naris | 22. External nares length | 13.52 | NA | 13.52 |
| External naris | 23. External nares width | 7.4 | NA | 7.4 |
| Maxilla | 24. Maxilla length | 43.56 | 43.56 | 37.99* |
| Maxilla | 25. Maxilla height | >27.75 | 27.75* | 27.02 |
| Lower jaw | 37. Jaw length total | 98.295 | 100.96 | 95.63 |
| Lower jaw | 38. Jaw length anterior to the coronoid process | 70.55 | 68.75 | 72.35 |
| Lower jaw | 39. Dentary height below teeth row | >19.47 | 19.47* | 19.39 |
| Lower jaw | 40. Height of coronoid process | >36.80 | 36.80* | 30.93 |
| Skull | 41. Shortest interorbital distance (width of the skull table) | >22.42 | NA | 11.21* |

**Table S2. Measurements of the axial elements of *Yamaceratops dorngobiensis*, MPC-D 100/553.** The measurement standards follow Tereshchenko (2015). An asterisk indicates measurement from a broken element as preserved.

| Element | Anterior height of centrum face | Posterior height of centrum face | Length of centrum | Midline height of the neural spine | Length of chevron |
| --- | --- | --- | --- | --- | --- |
| cd7 | NA | NA | NA | 8.94 | NA |
| cd13 | 5.98 | 5.62 | NA | 24.9 | NA |
| cd14 | 6 | 5.94 | 7.29 | 30.76 | 11.12 |
| cd15 | 5.44 | 5.86 | NA | 29.22 | 11.92 |
| cd16 | NA | 5.51 | 6.55 | 32.09 | 10.4 |
| cd17 | 6.27 | 6.18 | 6.18 | 32.47 | 10.81 |
| cd18 | 6.06 | 6.37 | 6.4 | NA | 11.04 |
| cd19 | NA | NA | 6.13 | NA | NA |

**Table S3. Measurements of the appendicular elements of *Yamaceratops dorngobiensis*, MPC-D 100/553.** The measurement standards follow Farke et al. (2013), Morschhauser et al. (2018b), and Słowiak et al. (2019). An asterisk indicates measurement from a broken element as preserved.

| Element | Description | Left | Right |
| --- | --- | --- | --- |
| Humerus | Total length | NA | 63.42* |
| Humerus | Minimum shaft circumference | NA | 23.02 |
| Humerus | Minimum shaft width | NA | 6.77 |
| Humerus | Maximum width of distal end | NA | 14.04 |
| Humerus | Length of deltopectoral crest | NA | 24.85 |
| Humerus | Distance from the distal end of the deltopectoral crest to the proximal end of the humerus | NA | 24.85* |
| Humerus | Distance from the distal end of the deltopectoral crest to the medial condyle of the humerus | NA | 36.41 |
| Humerus | Cranio-caudal diameter of the diaphysis | NA | 6.89 |
| Ulna | Shaft width in mid-length | NA | 5.62* |
| Radius | Total length | NA | 45.05* |
| Radius | Minimum shaft width | NA | 4.6 |
| Radius | Minimum shaft circumference | NA | 14.88 |
| Ilium | Total length | 81.09* | 77.28* |
| Ilium | Length of preacetabular process | 12.89* | 13.98* |
| Ilium | Length of postacetabular process | 40.96* | 42.87 |
| Ilium | Supracetabular height | 8.91* | 12.1 |
| Ischium | Total length | NA | 91.29* |
| Ischium | Shaft height posterior to the proximal plate | NA | 6.14 |
| Ischium | Anteroposterior length of iliac process | NA | 6.55 |
| Femur | Total length | 41.97* | 89.4* |
| Femur | Maximum width of the proximal end | 18.7 | 15.61* |
| Femur | Mid-shaft circumference | NA | 34.24 |
| Femur | Mid-shaft width | NA | 11.25 |
| Tibia | Total length | NA | 98.14 |
| Tibia | Transverse width of the distal end | NA | 13.07 |
| Tibia | Anteroposterior width of the proximal end | NA | 27.13* |
| Fibula | Total length | NA | 93.43 |
| Fibula | Maximum width of the proximal end | NA | 9.16 |
| Fibula | Maximum width of the distal end | NA | 4.4 |

**Table S4. Measurements of the left pes of *Yamaceratops dorngobiensis*, MPC-D 100/553.** The measurement standards follow Morschhauser et al. (2018b). An asterisk indicates measurement from a broken element as preserved.

| Digit | Description | Phalanx 1 | Phalanx 2 | Phalanx 3 | Phalanx 4 |
| --- | --- | --- | --- | --- | --- |
| I | Length | 18.52 | NA | NA | NA |
| I | Proximal width | 7.2 | NA | NA | NA |
| I | Distal width | 6.98* | NA | NA | NA |
| II | Length | 13.69* | 11.14* | NA | NA |
| II | Proximal width | 6.04 | 7.05 | NA | NA |
| II | Distal width | 7.93 | 7.66* | NA | NA |
| III | Length | 12.16 | 8.94 | 9.21 | 11.17* |
| III | Proximal width | 8.16* | 6.25* | 7.67* | NA |
| III | Distal width | 8.39* | 7.90* | 5.96* | 8.66* |

**3. Primary phylogenetic analysis**

**Modified scoring of *Yamaceratops* for the Arbour and Evans (2019) matrix**

11011011011?????1101110000010?0??11???001010100110021101010101001?01101110120000100011000101112?11011011?0?1?1?1101?0?110000101?11111000111?000100110001101000010000010??1110001210011011??????0??20141?1????????00??0???0[0 1]?000?0000011????01001000220102?11??111

**Changes in scorings applied to the Arbour and Evans (2019) matrix**

**Changes in scoring for *Yamaceratops* applied to the Arbour and Evans (2019) matrix**

3: ?->0; 5: ?->1; 6: ?->0; 10: ?->1; 11: ?->1; 17: ?->1; 18: ?->1; 19: ?->0; 23: ?->0; 26: ?->0; 27: ?->0; 28: ?->1; 29: ?->0; 31: ?->0; 46: ?->0; 49: ?->1; 95: ?->2; 103: ?->1; 126: ?->0; 127: ?->1; 172: [0 1 2]->1; 178: 0->1; 179: ?->0; 195: ?->2; 196: ?->0; 198: ?->4; 199: ?->1; 201: ?->1; 210: ?->0; 211: ?->0; 214: ?->0; 218: ?->0; 219: ?->[0 1]; 221: ?->0; 222: ?->0; 228: ?->0; 231: ?->1; 236: ?->0.

**Changes in scoring for *Protoceratops andrewsi* applied to the Arbour and Evans (2019) matrix**

198: ?->4.

**Changes in scoring for *Aquilops* applied to the Arbour and Evans (2019) matrix (ontogenetically variable characters)**

8: 1->?; 32: 0->?; 58: 0->?; 98: 0->?; 177: 2->?.

**Changes in scoring for ‘*Graciliceratops*’ applied to the Arbour and Evans (2019) matrix (ontogenetically variable characters)**

58: 1->?; 59: 0->?.

**Ontogenetically variable characters from the Arbour and Evans (2019) matrix.** Newly noted characters in asterisk.

8. Rostral ventral (buccal) process absent (0) or present (1).

32*. Nasal shape: straight lateral aspect and flat (0), dorsoventrally bowed (1), or ornamented (2).

53. Rugosity on the jugal, dentary and surangular absent (0), present, weakly developed (1), present, strongly developed (2).

58. Epijugal ossification absent (0) or present (1).

59. Epijugal position on jugal: along dorsal edge of horn (epijugal trapezoidal) (0) or capping end of horn (epijugal conical) (1).

98. Degree of incisure of frontal fossae/parieto-frontal contact: incisures absent (0); shallow (1); deeper (2); fronto-parietal depression (3); invaginated by fontanelle, secondary skull roof present (4).

128*. Predentary less than two-thirds of dentary length (0) or equal to or more than two-thirds of dentary length (1).

162. Caudal edge of the angular horizontal or gently sloping to contact the surangular (0) formed by a caudally facing triangular surface or bearing a step (1).

177. Teeth without distinct median primary ridge (0) or with very weak and wide median ridge on at least some maxillary teeth (1) or all maxillary and dentary teeth with distinct primary ridge (2).

**Time calibration** **from the Arbour and Evans (2019) matrix.**

Some of the ages used to calibrate the cladogram from the Morschhauser et al. (2018c) matrix have been updated. The changed taxa and age range (FAD-LAD) are as follows:

*Chaoyangsaurus*: 168.3-166.1 -> 154-137 Ma (Xu et al., 2012);

*Liaoceratops*:126.8-119 -> 125.76-124.12 Ma (Zhong et al., 2021);

*Ischioceratops*: 76.38-66 -> 77.3-73.5 Ma (An et al., 2016);

*Zhuchengceratops*: 76.38-73.5 -> 77.3-73.5 Ma (An et al., 2016);

*Psittacosaurus sinensis*: 129.4-125 -> 121-119 Ma (Zhou, 2010);

*Archaeoceratops*: 113-100.5 -> 112-105 Ma (Zheng et al., 2021);

*Auroraceratops*: 107-100.5 -> 112-105 Ma (Zheng et al., 2021).

*Ferrisaurus*: 68.2-67.2 Ma (newly added; Arbour and Evans, 2019)

The earliest fossil record of ceratopsian dinosaurs appears to be two Jurassic taxa, *Yinlong* and *Hualianceratops*, both from the Upper Jurassic (Oxfordian) Shishugou Formation in layers dating between 159.7+/-0.3 and 162.2+/-0.2 Ma (Xu et al., 2006; Han et al., 2015; 2016). Although Zhao et al. (1999) suggested a Middle Jurassic (Bathonian) age for the Tuchengzi Formation where *Chaoyangsaurus* was recovered, recent dating has suggested a Late Jurassic to Early Cretaceous age (154 to 137 Ma by Xu et al., 2012; 147 to 136 Ma by Zhang et al., 2009). Even the Tiaojishan Formation that unconformably underlies the Tuchengzi Formation is likely mostly Upper Jurassic, with a lower boundary of 165 Ma and upper boundary of 156-153 Ma (Zhang et al., 2008). The Houcheng Formation in Hebei Province, where *Xuanhuaceratops* was discovered (Zhao et al., 2006), is laterally equivalent to the Tuchengzi Formation in Liaoning Province (Wang, 1998; Xu et al., 2012), and hence of the same age (Xu et al., 2012).


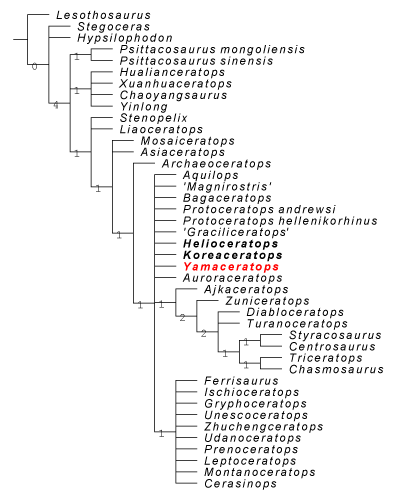


**Figure S4. Phylogenetic relationships of *Yamaceratops dorngobiensis* among ceratopsians using the Arbour and Evans (2019) matrix with revised scorings, without pruning of taxa.** Strict consensus tree constructed by using the character matrix of Morschhauser et al. (2018c) (as iterated by Arbour and Evans (2019) with the addition of *Ferrisaurus*) with updated scorings for *Yamaceratops*. *Yamaceratops* is highlighted in red color with *Helioceratops* and *Koreaceratops* in bold. Numbers at each node indicate Bremer support values.


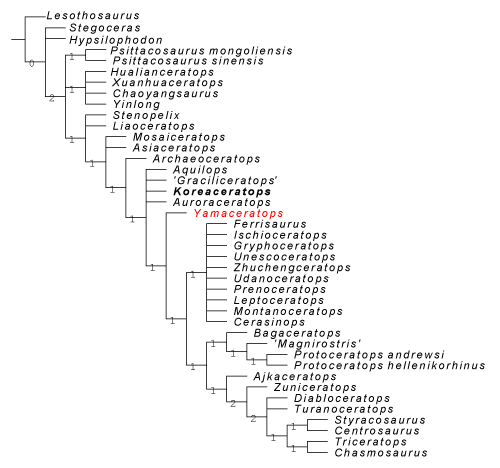


**Figure S5. Phylogenetic relationships of *Yamaceratops dorngobiensis* among ceratopsians using the Arbour and Evans (2019) matrix with revised scorings, with *Helioceratops* removed.** Strict consensus tree constructed by using the character matrix of Morschhauser et al. (2018c) (as iterated by Arbour and Evans (2019) with the addition of *Ferrisaurus*) with updated scorings for *Yamaceratops*. *Yamaceratops* is highlighted in red color with *Koreaceratops* in bold. Numbers at each node indicate Bremer support values.

**4. Supplementary phylogenetic analysis**

**Modified scoring of *Yamaceratops* for the Yu et al. (2020) matrix**

11102?001211110?11???????????????100????11100????1111??00000???????????????100?000?????????101100??0110000?01000000??12???????00100?1??????????1?00?????0????????????????????????????????????????????01??0?1???10?1??0110000100??1110??110100?11000100?11000???01000111010??1000210111100?0110010???????0??0???2?0?13???????????????????0???????000???100?0?00

**Changes in scoring for *Yamaceratops* applied to the Yu et al. (2020) matrix**

3: ?->1; 4: ?->0; 10: ?->2; 12: ?->1; 13: ?->1; 14: ?->1; 15: ?->0; 17: ?->1; 18: ?->1; 34: ?->1; 35: ?->0; 36: ?->0; 41: ?->1; 42: ?->1; 43: ?->1; 44: ?->0; 45: ?->0; 52: ?->1; 53: ?->1; 56: ?->0; 60: ?->0; 76: ?->1; 77: ?->0; 78: ?->0; 92: ?->1; 93: ?->0; 94: ?->1; 95: ?->1; 96: ?->0; 100: ?->0; 101: ?->1; 102: ?->1; 103: ?->0; 104: ?->0; 105: ?->0; 106: ?->0; 109: ?->1; 110: ?->0; 111: ?->0; 112: ?->0; 113: ?->0; 114: ?->0; 119: ?->2; 144: ?->1; 146: ?->0; 147: ?->0; 215: ?->1; 229: ?->0; 232: ?->1; 234: ?->0; 236: ?->0; 237: ?->0; 243: ?->0; 246: ?->0; 250: ?->0; 253: 0->?; 258: ?->0; 266: ?->0; 273: 1->2; 274: 0->1; 277: ?->1; 284: ?->1; 285: ?->1; 304: ?->2; 306: ?->; 309: ?->3; 329: ?->0; 337: ?->0; 338: ?->0; 339: ?->0; 343: ?->1; 344: ?->0; 345: ?->0; 347: ?->0.

**Phylogenetic relationships of *Yamaceratops dorngobiensis* among ceratopsians using the Yu et al. (2020) matrix as modified from the Knapp et al. (2018) matrix**

For the confirmation of the phylogenetic position of *Yamaceratops*, a second analysis was conducted using the character matrix of Knapp et al. (2018) as iterated by Yu et al. (2020), with only scorings for *Yamaceratops* revised, for 71 taxa with 350 unordered characters using the new technology search algorithm (Fig. S6). The Knapp et al. (2018) matrix encompasses most of the ceratopsid taxa in addition to basal ceratopsians, as opposed to the Morschhauser et al. (2018c) matrix, which is exclusively focused on basal neoceratopsians, with few ceratopsids. However, because the Knapp et al. (2018) matrix focused on ceratopsids, many of the characters are on the elaborated frills and horns that are only applicable in ceratopsids. Testing the phylogenetic hypothesis for *Yamaceratops* with the Yu et al. (2020) matrix, the resulting topology is consistent with that of Yu et al. (2020), with paraphyletic Chaoyangsauridae, Psittacosauridae, and Protoceratopsidae. Noticeable differences in the neoceratopsian topology are *Asiaceratops* recovered as a leptoceratopsid and *Yamaceratops* recovered as the sister taxon to the clade consisting of leptoceratopsids and coronosaurs in the new analysis, both of which were recovered as more basal in the previous result. Compared with our results from the Arbour and Evans (2019) matrix (reiteration of the Morschhauser et al. (2018c) matrix), our new cladogram from the Yu et al. (2020) matrix (reiteration of the Knapp et al. (2018) matrix) recovered a more basal position for *Aquilops* and a more derived position for *Asiaceratops* and ‘*Graciliceratops*.’ Given that these three taxa are known only from juvenile specimens, it seems likely that their positions are subject to changes when ontogenetic variations are considered in the character scorings in the Yu et al. (2020) matrix. Considering juvenile features in scoring the characters from the Knapp et al. (2018) matrix is more complicated because the ontogenetic trajectories of basal neoceratopsians differ from those of ceratopsids, with heterochronic development of derived features (Prieto-Márquez et al., 2020) and should be further studied in the future.


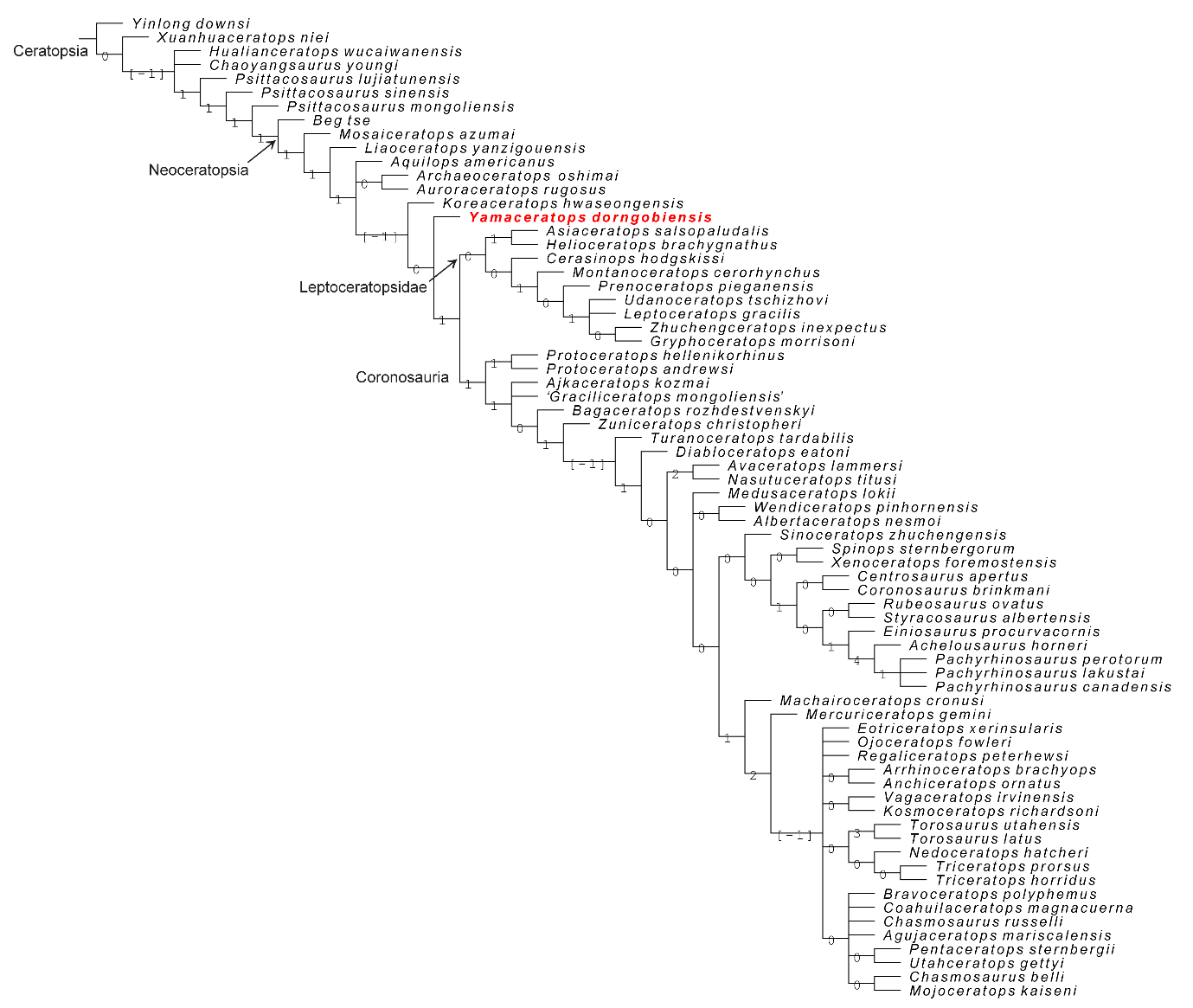


**Figure S6. Phylogenetic relationships of *Yamaceratops dorngobiensis* among ceratopsians using the Yu et al. (2020) matrix.** Strict consensus tree constructed by using the character matrix of Yu et al. (2020) (iteration of the Knapp et al. (2018) matrix) with updated scorings for *Yamaceratops dorngobiensis*. *Yamaceratops dorngobiensis* is highlighted in red color. Numbers at each node indicate Bremer support values.

**5. Supplementary references**

Arbour, V. M., & Evans, D. C. (2019). A new leptoceratopsid dinosaur from Maastrichtian-aged deposits of the Sustut Basin, northern British Columbia, Canada. *PeerJ*, *7*, e7926.

An, W., Kuang, H. W., Liu, Y. Q., Peng, N., Xu, K. M., Xu, H., ... & Zhang, Y. X. (2016). Detrital zircon dating and tracing the provenance of dinosaur bone beds from the Late Cretaceous Wangshi Group in Zhucheng, Shandong, East China. Journal of Palaeogeography, 5(1), 72-99.

Han, F., Forster, C. A., Clark, J. M., & Xu, X. (2015). A new taxon of basal ceratopsian from China and the early evolution of Ceratopsia. *PloS one*, *10*(12), e0143369.

Han, F.-L., Forster, C. A., Clark, J. M., & Xu, X. (2016). Cranial anatomy of *Yinlong downsi* (Ornithischia: Ceratopsia) from the Upper Jurassic Shishugou Formation of Xinjiang, China. *Journal of Vertebrate Paleontology*, *36*(1), e1029579.

Knapp, A., Knell, R. J., Farke, A. A., Loewen, M. A., & Hone, D. W. (2018). Patterns of divergence in the morphology of ceratopsian dinosaurs: sympatry is not a driver of ornament evolution. *Proceedings of the Royal Society B: Biological Sciences*, *285*(1875), 20180312.

Morschhauser, E. M., You, H., Li, D., & Dodson, P. (2018c). Phylogenetic history of *Auroraceratops rugosus* (Ceratopsia: Ornithischia) from the Lower Cretaceous of Gansu Province, China. *Journal of Vertebrate Paleontology*, *38*(sup1), 117-147.

Prieto‐Márquez, A., Garcia‐Porta, J., Joshi, S. H., Norell, M. A., & Makovicky, P. J. (2020). Modularity and heterochrony in the evolution of the ceratopsian dinosaur frill. *Ecology and Evolution*, *10*(13), 6288-6309.

Wang, X., Wang, Y., Wang, Y., Xu, X., Tang, Z., Zhang, F., ... & Hao, Z. (1998). Stratigraphic sequence and vertebrate-bearing beds of the lower part of the Yixian Formation in Sihetun and neighboring area, western Liaoning, China. *Vertebrata PalAsiatica*, *36*(02), 81.

Xu, X., Forster, C. A., Clark, J. M., & Mo, J. (2006). A basal ceratopsian with transitional features from the Late Jurassic of northwestern China. *Proceedings of the Royal Society B: Biological Sciences*, *273*(1598), 2135-2140.

Xu, H., Liu, Y. Q., Kuang, H. W., Jiang, X. J., & Peng, N. (2012). U–Pb SHRIMP age for the Tuchengzi Formation, northern China, and its implications for biotic evolution during the Jurassic–Cretaceous transition. Palaeoworld, 21(3-4), 222-234.

Yu, C., Prieto-Marquez, A., Chinzorig, T., Badamkhatan, Z., & Norell, M. (2020). A neoceratopsian dinosaur from the early Cretaceous of Mongolia and the early evolution of ceratopsia. *Communications biology*, *3*(1), 1-8.

Zhang, H., Wang, M., & Liu, X. (2008). Constraints on the upper boundary age of the Tiaojishan Formation volcanic rocks in West Liaoning-North Hebei by LA-ICP-MS dating. *Chinese Science Bulletin*, *53*(22), 3574-3584.

Zhang, H., Wei, Z., Liu, X., & Li, D. (2009). Constraints on the age of the Tuchengzi Formation by LA-ICP-MS dating in northern Hebei-western Liaoning, China. *Science in China Series D: Earth Sciences*, *52*(4), 461-470.

Zhao, X., Cheng, Z., & Xu, X. (1999). The earliest ceratopsian from the Tuchengzi Formation of Liaoning, China. *Journal of Vertebrate Paleontology*, *19*(4), 681-691.

Zhao, X., Cheng, Z., Xu, X., & Makovicky, P. J. (2006). A new ceratopsian from the Upper Jurassic Houcheng Formation of Hebei, China. *Acta Geologica Sinica‐English Edition*, *80*(4), 467-473.

Zheng, D., Wang, H., Li, S., Wang, B., Jarzembowski, E. A., Dong, C., ... & Zhang, H. (2021). Synthesis of a chrono-and biostratigraphical framework for the Lower Cretaceous of Jiuquan, NW China: Implications for major evolutionary events. Earth-Science Reviews, 213, 103474.

Zhong, Y., Huyskens, M. H., Yin, Q. Z., Wang, Y., Ma, Q., & Xu, Y. G. (2021). High-precision geochronological constraints on the duration of ‘Dinosaur Pompeii’ and the Yixian Formation. National Science Review, 8(6), nwab063.

Zhou, C. F. (2010). A possible azhdarchid pterosaur from the Lower Cretaceous Qingshan Group of Laiyang, Shandong, China. Journal of Vertebrate Paleontology, 30(6), 1743-1746.
